# Genotoxicity and Epigenotoxicity of Carbazole-Derived Molecules on MCF-7 Breast Cancer Cells

**DOI:** 10.3390/ijms22073410

**Published:** 2021-03-26

**Authors:** Claudio Luparello, Ilenia Cruciata, Andreas C. Joerger, Cory A. Ocasio, Rhiannon Jones, Raysa Khan Tareque, Mark C. Bagley, John Spencer, Martin Walker, Carol Austin, Tiziana Ferrara, Pietro D′Oca, Rossella Bellina, Rossella Branni, Fabio Caradonna

**Affiliations:** 1 Department of Biological Chemical and Pharmaceutical Sciences and Technologies, University of Palermo, Viale delle Scienze, Edificio 16, 90128 Palermo, Italy; ilenia.cruciata@unipa.it (I.C.); ferrarati@libero.it (T.F.); docapietro22@gmail.com (P.D.); bellinarossella@gmail.com (R.B.); brannyrossy@gmail.com (R.B.); 2 Institute of Pharmaceutical Chemistry, Goethe University, 60438 Frankfurt am Main, Germany; joerger@pharmchem.uni-frankfurt.de; 3 Buchmann Institute for Molecular Life Sciences, Structural Genomics Consortium (SGC), 60438 Frankfurt am Main, Germany; 4 Department of Chemistry, School of Life Sciences, University of Sussex, Falmer, Brighton BN1 9QJ, UK; tony.ocasio@crick.ac.uk (C.A.O.); rhiannon.n.jones2312@gmail.com (R.J.); raysa_khan@hotmail.com (R.K.T.); M.C.Bagley@sussex.ac.uk (M.C.B.); 5 Eurofins Integrated Discovery UK Ltd., Fyfield Business & Research Park, Fyfield Road, Ongar, Essex CM5 0GS, UK; martin.walker@selcia.com (M.W.); carol.austin@selcia.com (C.A.)

**Keywords:** genomic instability, carbazole derivatives, epigenetics, DNA methylation, breast cancer

## Abstract

The carbazole compounds **PK9320** (1-(9-ethyl-7-(furan-2-yl)-9H-carbazol-3-yl)-N-methylmethanamine) and **PK9323** (1-(9-ethyl-7-(thiazol-4-yl)-9H-carbazol-3-yl)-N-methylmethanamine), second-generation analogues of **PK083** (1-(9-ethyl-9H-carbazol-3-yl)-N-methylmethanamine), restore p53 signaling in Y220C p53-mutated cancer cells by binding to a mutation-induced surface crevice and acting as molecular chaperones. In the present paper, these three molecules have been tested for mutant p53-independent genotoxic and epigenomic effects on wild-type p53 MCF-7 breast adenocarcinoma cells, employing a combination of Western blot for phospho-γH2AX histone, Comet assay and methylation-sensitive arbitrarily primed PCR to analyze their intrinsic DNA damage-inducing and DNA methylation-changing abilities. We demonstrate that small modifications in the substitution patterns of carbazoles can have profound effects on their intrinsic genotoxic and epigenetic properties, with **PK9320** and **PK9323** being eligible candidates as “anticancer compounds” and “anticancer epi-compounds” and **PK083** a “damage-corrective” compound on human breast adenocarcinoma cells. Such different properties may be exploited for their use as anticancer agents and chemical probes.

## 1. Introduction

Genotoxicity, as defined by toxicological genetics and mutagenesis, describes the ability of chemical agents to induce modifications to the DNA nucleotide sequence or double helix structure of an organism, damaging its genetic information, thereby causing mutations that can lead to the onset of cancer [1]. DNA damage is one of the most dangerous threats to cellular homeostasis, and, for this reason, the cellular response to this event is immediate and vigorous. This DNA-damage response (DDR) encompasses numerous metabolic pathways, and its defects leave cells susceptible to genomic instability, predispose to further genomic errors and, lastly, transformation.

Since the early discovery of the antibiotic properties of the carbazole alkaloids ellipticine and murrayanine extracted from *Ochrosia elliptica* Labill and *Murraya koenigii* Spreng, respectively [2,3], natural carbazoles and their synthetic derivatives have been extensively studied and incorporated as key structural motifs in a number of approved drugs acting against a wide spectrum of diseases, including cancer, microbial infections and neurological disorders [4,5,6]. However, carbazoles have also been shown to induce epigenetic anomalies, such as changes in DNA methylation status, and also to exert genotoxic effects. To cite some examples, exposure to mahanine, a carbazole alkaloid from *Micromelum minutum*, was found to be responsible for the negative modulation of the cellular levels of selected DNA methyltransferases in prostate tumor cells, thereby restoring the expression of the epigenetically silenced tumor suppressor gene *RASSF1A* (Ras Association Domain Family Member 1). Moreover, SH-I-14 was shown to determine the promoter demethylation-induced reactivation of tumor-suppressor gene expression in triple-negative breast cancer cells. The epigenetic impact of 2,7-dibromocarbazole and 6-formylindolo [3,2-b]carbazole has been also acknowledged. In particular, the former molecule was proven to demethylate *Ang2* (angiopoietin 2) gene promoter and potentiate its expression, resulting in deficient angiogenesis by endothelial cells. The latter one, a ligand of the aryl hydrocarbon receptor, was found to repress the expression of the *CYP1A1* (aryl hydrocarbon hydroxylase) gene in hepatoma cells by targeting DNA methyltransferase 3A, which led to impairment of the cellular detoxification system [7,8,9,10]. Furthermore, Valovičová et al. [11] demonstrated the occurrence of significant levels of DNA strand breaks, micronuclei, and DNA adducts after exposure of human keratinocytes to 7H-dibenzo[*c,g*]carbazole, and Zhanataev et al. [12] reported genotoxic effects of two carbazole-derived xenomycins, PLX01107 and PLX01008, initially developed as potential antifungal agents. Although the genotoxic and epigenotoxic side effects of carbazoles can be an issue when administered to healthy tissues, thus limiting the use of the compounds in clinical practice, these properties make them promising “anticancer (epi)drugs” efficacious as preventative and/or treatment agents in a combinatorial molecular therapy against metastatic cancer histotypes.

**Figure 1:**
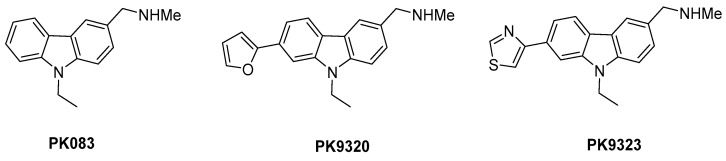
Chemical structures of the carbazole derivatives **PK083**, **PK9320** and **PK9323**.

Carbazole derivatives have also been used as chemical chaperones to reactivate a thermolabile p53 cancer mutant. The p53 cancer hotspot mutation Y220C greatly destabilizes the p53 DNA-binding domain, which unfolds and aggregates at body temperature, resulting in the loss of p53 tumor suppressor function [13]. Structural studies have further shown that the mutation creates a druggable cavity on the surface that can be targeted by small molecules. **PK083** (Figure 1) is a carbazole-based small molecule selected as a starting scaffold to stabilize and reactivate the Y220C mutant [14]. Structure-guided design then led to second-generation analogues, including **PK9320** and **PK9323**, which bound the mutant with higher affinity and were more potent in stabilizing and reactivating the mutant than the parent compound (Figure 1). Importantly, those second-generation carbazoles restored p53 signaling in the liver cancer cell line HUH-7 bearing a homozygous Y220C mutation, with potential future application in personalized cancer therapy [15,16].

Here, we were interested in gaining insights into potential mutant p53-independent genotoxic or epigenetic effects of this carbazole series at high compound concentrations, using compounds **PK083**, **PK9320** and **PK9323** as examples. We have therefore employed a combination of Western blot, Comet assay and methylation-sensitive arbitrarily primed PCR techniques to analyze their DNA damage-inducing and DNA methylation-changing abilities in wild-type p53 MCF-7 breast adenocarcinoma cells.

## 2. Results

In order to elucidate the potential genotoxic mechanism exhibited by **PK083**, **PK9320** and **PK9323**, histone H2AX phosphorylation and Comet assays were conducted after exposure of MCF-7 breast cancer cells to each of the molecules. It is known that, in response to ionizing radiation or DNA-damaging chemotherapeutic agents, DNA double-strand breaks (DSB) are generated that rapidly result in the phosphorylation of the histone H2A variant H2AX [17]. Because the phosphorylation of H2AX at Ser139 (γ-H2AX) is abundant, fast and correlates with DSB, it represents a very sensitive marker of DNA damage and the subsequent repair, and is therefore a commonly used test to identify genotoxic compounds [18].

**Figure 2:**
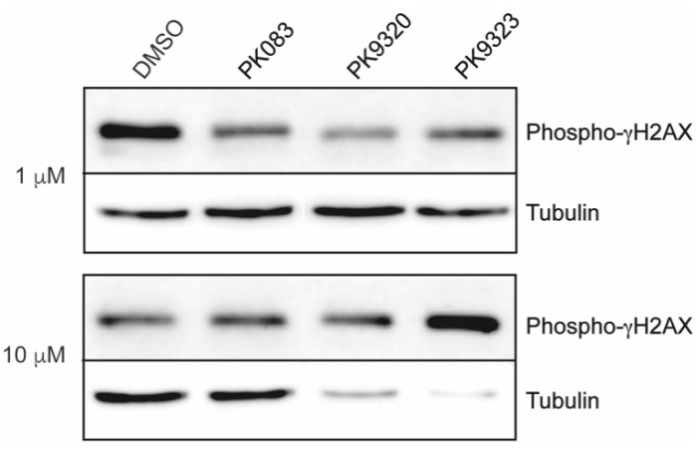
MCF-7 cells were treated with DMSO control or an indicated concentration of compound. After 20 h, cells were harvested, lysed and probed for DNA damage by Western blot analysis using a phosphorylated γH2AX antibody. Western blot analysis of tubulin was used as a loading and cell viability control.

**Figure 3:**
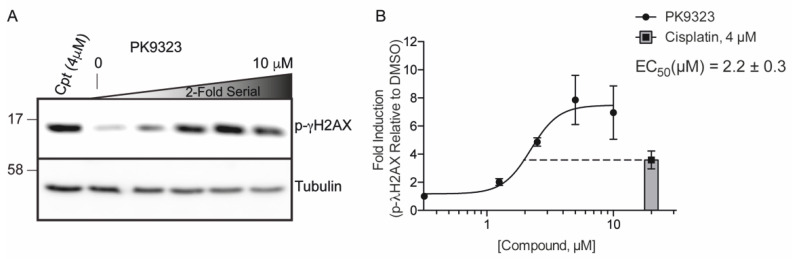
MCF-7 cells were treated with 2-fold serial dilutions of PK9323 from 10 down to 0 μM. After 20 h, cells were harvested, lysed and probed for DNA damage by Western blot analysis (A) using a phosphorylated γH2AX antibody. Western blot analysis of tubulin was used as a loading and cell viability control. B) Dose-response curve for γH2AX phosphorylation after treatment of MCF-7 cells with **PK9323** for 20 h; DMSO was used as a no compound control. The EC_50_ of **PK9323** is 2.2 ± 0.3 μM and cisplatin was tested in parallel at 4 μM as a reference DNA lesion-inducing drug. The error bars correspond to the SD of three independent measurements.

As shown in Figure 2, **PK9323,** at 10 μM concentration, led to enhanced γH2AX phosphorylation compared with the untreated control, correlating with more extensive DNA damage. In contrast, **PK083** and **PK9320** were not found to affect the level of histone phosphorylation and were genotoxically inactive in that aspect. However, **PK9320** showed a cytotoxic effect, as evidenced by the drastic decrease in tubulin levels, suggesting cell death or inhibition of cell growth. The exposure–response relationship was further investigated, identifying **PK9323** as relatively highly genotoxic, with an EC_50_ of 2.2 μM, which exceeds that of cisplatin (EC_50_ = 4 μM), as shown in Figure 3. The optical densities of the Western blot bands shown in both figures can be found in Figure S1.

To gain more insight into this latter aspect, MCF-7 cells were exposed to **PK9323**, and its effect on cell morphology and growth rate was analyzed by microscopic observations and direct cell counting also comparing it to those exerted by **PK083** and **PK9320**. No significant morphological change indicative of cell suffering or death could be observed with all treatments, even when exposing cells to the maximum concentration of the compounds (Figure S2).

**Figure 4:**
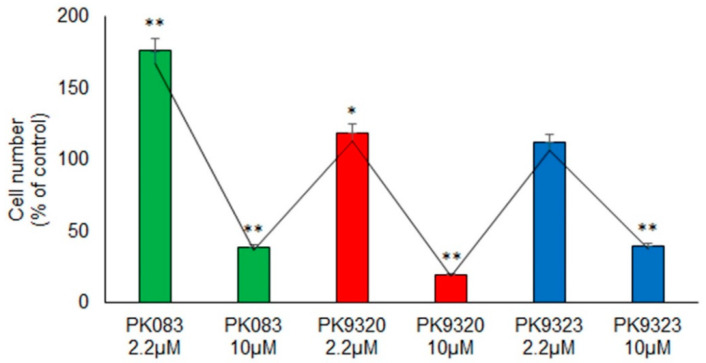
Effect of the carbazole derivatives on the number of MCF-7 cells after 20 h of exposure. Cell numbers were measured by direct counting in a Bürker chamber after trypsinization. Error bars correspond to the SEM of three independent measurements. The black line represents the IQR values. * = *p* < 0.05 vs. control; ** = *p* << 0.001 vs. control.

The numbers of control and treated cells were then evaluated by counting in a haemocytometer, and the results confirm that cell exposure to 10 μM **PK9320** appeared to drastically decrease the cell count to circa 20% vs. control. Interestingly, also in the presence of **PK083** and **PK9323** at 10 μM concentrations, the amount of cells decreased, although to a lesser degree than with **PK9320** (ca. 39% and 40% vs. control, respectively). At a lower concentration of 2.2 μM, **PK9320** and **PK9323** exerted only a negligible effect on cell growth (ca. 119% and 112% vs. control, respectively), whereas, conversely, **PK083** induced an average 76% increase in the cell number with respect to the untreated samples (Figure 4).

**Figure 5:**
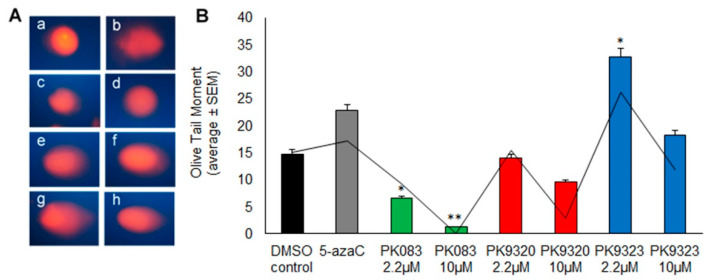
Effect of the carbazole derivatives on the occurrence of genomic DNA lesions in MCF-7 cells after 20 h of exposure. (**A**) Representative micrographs of comet images obtained from MCF-7 cells exposed to DMSO vehicle as control (a), 10 μM 5-azaC (b) and **PK083** (c,d), **PK9320** (e,f), or **PK9323** (g,h) at 2.2 μM (c,e,g) or 10 μM concentrations (d,f,h), stained with Gel Red DNA Dye and observed with a Nikon Microphot SA fluorescence microscope. Each spot corresponds to the DNA of an individual cell: when round it represents the intact DNA which is a large macromolecule that poorly migrates in the electrophoretic field, when “comet shaped” it is symptomatic of DNA breaks rendering the molecule small enough to move in the gel. Microscopic magnification = 400× *g*. (**B**) Histogram showing the induced genomic DNA damage evaluated as OTM by Comet assay. OTM is the product of the percentage of total DNA in the tail and the distance between the bulk of head and tail regions. The number of scored cells was 50 for each experimental condition. The black line represents the IQR values. * = *p* < 0.05 vs. control; ** = *p* << 0.001 vs. control.

The possible occurrence of genomic DNA lesions induced by the carbazole compounds was quantitatively evaluated by Comet assay in terms of average Olive Tail Moment (OTM), indicative of persistent single- and double-strand breaks. Preliminary data showed that the Student′s *t* test did not reveal significant differences between the OTM values obtained from the untreated and the control DMSO-vehicle-only exposed cultures (data not shown), thereby ensuring that the vehicle did not influence the experimental data. The data shown in Figure 5 indicate that the OTM of the DNA methyltransferase inhibitor 5-azacitidine (5-azaC)-treated cells was not significantly different from that of the DMSO control, confirming that no toxic effect was exerted by the methylation inhibitor on the cells under the conditions used. This suggested a very low probability of the onset of clonal selection, an important premise for the validation of the subsequent methylation analyses. Moreover, **PK9320** and **PK083**, at both concentrations tested, appeared unable to cause damage to the genomic DNA, with **PK083** rather exerting a protective action, as shown by the OTM value significantly lower than that of untreated cells. Conversely, as expected from the results of the histone γH2AX phosphorylation assay, **PK9323** induced a considerable degree of genomic DNA damage at 2.2 μM concentration, whereas at 10 μM concentration the difference with the DMSO control was not statistically significant, probably due to a clonal selection phenomenon leading to the preferential survival of one or more clones better equipped to repair DNA breakages.

**Figure 6:**
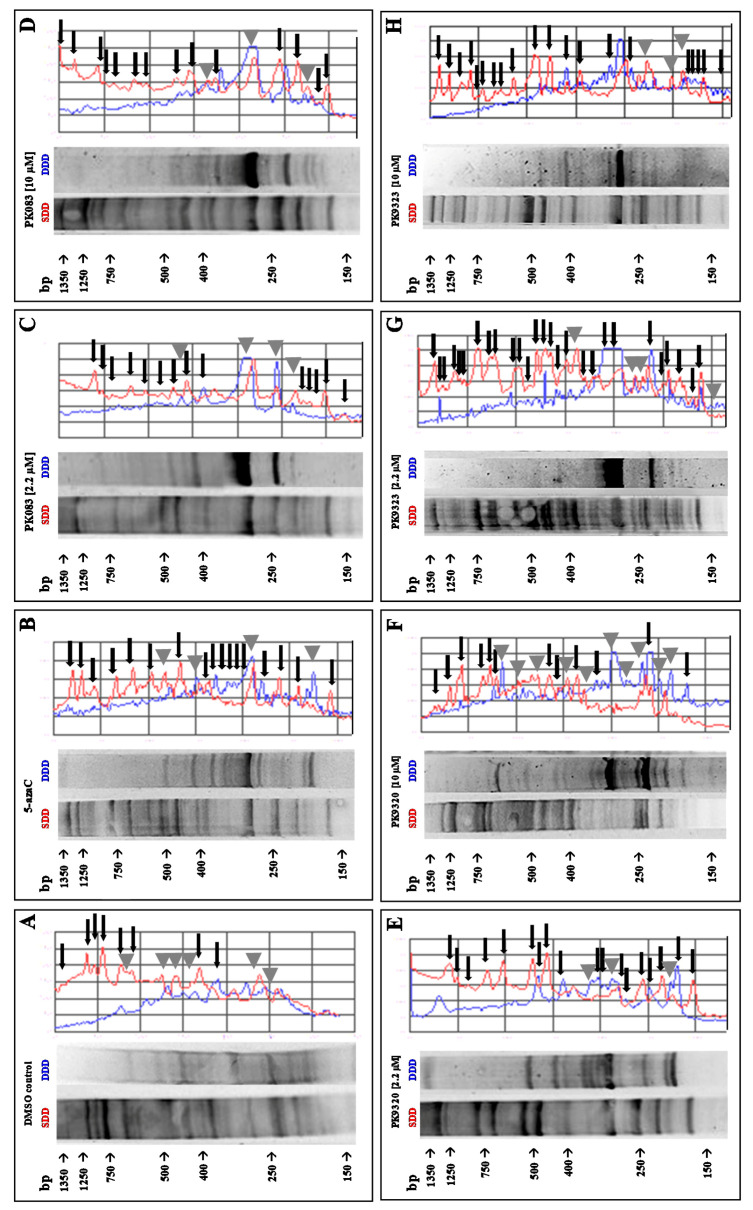
Analysis of genome-wide DNA methylation status. Representative MeSAP fingerprintings and corresponding densitometry profiles of single-digested DNA (SDD) and double-digested DNA (DDD) samples from DMSO vehicle (**A**) and 5-azaC- (**B**), PK083- (**C**,**D**), PK9320 (**E**,**F**) or PK9323-treated MCF-7 cells (**G**,**H**) at 2.2 (**C**,**E**,**G**) and 10 µM concentrations (**D**,**F**,**H**). Changes of the DNA band patterns are indicated with black arrows (A/D) or grey arrowheads (A/I).

**Figure 7:**
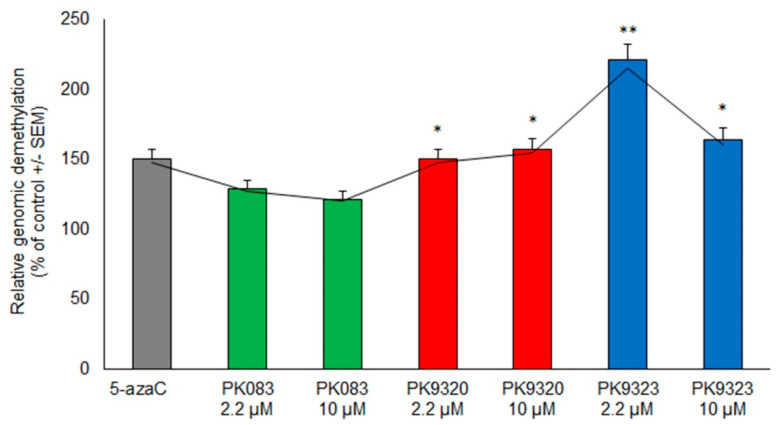
Effect of the carbazole derivatives on the global methylation pattern of MCF-7 cells after 20 h of exposure. The histogram shows the percentage of genomic demethylation of exposed MCF-7 cells vs. controls, as calculated by the number of band pattern variations. The black line represents the IQR values. * = *p* < 0.05; ** = *p* < 0.001.

In a last set of assays, we investigated in more detail the epigenetic modulation carried out by the three compounds through the analysis of the global methylation status of the genomic DNA. For this purpose, MeSAP-PCR experiments were carried out on single- (SDD) and double-digested DNA (DDD) samples obtained from control and **PK083**-, **PK9320**- or **PK9323**-treated MCF-7 cells at 2.2 and 10 μM concentrations. The DNA fingerprintings were analyzed by densitometric scanning, and the numbers of variations, in terms of appearing/disappearing (A/D) and attenuation/intensification (A/I) of the band patterns in the SDD vs. DDD preparations, were determined (Figure 6). The obtained data are summarized in Table S1 and shown in the histogram in Figure 7 and indicated that 20 h treatments with both concentrations of **PK9320** and **PK9323** were effective in modifying the global methylation pattern of cancer cell DNA, as shown by the different number, intensity and size of the bands in the matched control and exposed samples; in particular, the most numerous changes were observed after cell exposure to 2.2 μM **PK9323**. Conversely, the difference between the modulatory activities of **PK083** and the vehicle alone was not significant.

As expected, the results obtained with 5-azaC-treated cells were significantly different from those of the control. This finding, in the absence of clonal selection towards an epigenetically unbalanced cell population, demonstrates that MCF-7 cells are sensitive to the modulation of DNA methylation mediated by the “DNA methyltransferase-S adenosylmethionine (DNMT-SAM)” system, the main pathway by which genomic DNA methylation is ensured.

All three carbazoles in this study have been investigated in pharmacokinetic (PK) studies, notably looking at cytotoxicity in HepG2 liver cancer cells. Further studies looked at aqueous solubility and all compounds had acceptable values at pH 7 of >100 mM. Measurement of clearance (compound metabolic stability), in mouse microsomes, showed **PK9323** to have a reasonable half-life (>1 h), coupled with a relatively low clearance with around 3% unbound drug in plasma protein binding studies (PPB). **PK9320** had the shortest half-life and highest clearance of the three compounds studied; moreover, it had a very low % PPB. Concerning compound cytotoxicity, the following results were obtained: **PK083** (23 μM); **PK9320** (4.8 μM); **PK9323** (1.1 μM), with **PK083** showing mild and **PK9323** high cytotoxicity in this assay (Table S2). These data appear to align well with the other studies outlined above.

## 3. Discussion

It is widely acknowledged that members of the family of carbazole-based molecules, either natural or synthetic, represent promising compounds with the ability to act as DNA intercalating agents and to interfere with the activity of crucial enzymes, such as topoisomerases and telomerases [19]. The topology of DNA and the maintenance of telomere length are two crucial endpoints whereby cells, physiologically, especially transformed ones, make nicks in the DNA and manage them, thus exposing themselves to the risk of creating free DNA ends prone to produce chromosomal aberrations that can initiate/promote tumorigenesis or induce cell death [20,21].

Moreover, some carbazole-derived molecules affect DNA methylation at specific CpG islands [9]. Because of this property, they currently represent the mainstay of treatment decisions for leukemia patients who are not suitable for intensive chemotherapy [22], also because they can be safely metabolized by CYP enzymes [23]. It is also important to remember that the expression of some members of the cytochrome P450 family, which is composed of several genes [24], is regulated by DNA methylation [25].

Here, we have analyzed the DNA damage-inducing potential and methylation-changing capability of three carbazoles, PK083, PK9320, and PK9323, in human breast adenocarcinoma cells (MCF-7) to evaluate their potential use as “anticancer (epi)drugs” in a combinatorial molecular therapy [26], in addition to their reported action as Y220C mutant p53 reactivators [15,16].

We have chosen to investigate the genotoxic potency of the three molecules, quantitatively and qualitatively, by performing the histone γH2AX phosphorylation assay and the Comet assay. Using these two complementary techniques enabled us to look at two facets of the same kind of DNA damage: from the molecular point of view and from the cellular one. In fact, the extent of H2AX phosphorylation correlates with the number of regions with DNA DSB, which are involved in the recruitment of DNA repair factors; at the chromatin level, a DNA damage site, even in a transformed cell and within certain limits, can still be repaired and its effect be silenced or underestimated. Also for this reason, this assay was called “a surrogate marker” of DSB study [27]. In contrast, the alkaline Comet assay, a useful technique for detecting the cyto-electrophoretic migration of nuclear DNA with single- and double-strand breaks, visually and quantitatively shows DNA damage at the cellular/nuclear level originating from several sources, even in the long term [28], providing an overall picture of an entire population of cells “sensitively and precisely”, as was more recently reported [29]. Hence, both points of view were considered to better characterize the genotoxic potency and to hypothesize more precisely on the mechanism of action of the inducing molecules.

An innovative feature of this study is its epigenomic aspect. The in vitro/in vivo study of the epigenetic effects of carbazole derivatives is a relatively new field, and only a few publications have addressed this topic to date (A PubMed search in February 2021, with “carbazole derivative epigenetic” as key words retrieved only 14 hits). Given that the modulation of genomic DNA methylation is one of the crucial epigenetic modifications in the oncogenic process and a biomarker implicated in various human pathologies, including cancer [30], we also wanted to evaluate the capacity of the three molecules to act as epigenomic modulators.

Taking the obtained results and the literature data into consideration, the findings reported here allow us to make the following comments:

(1) First of all, it must be highlighted that the Comet assay performed on 5-azaC-treated cells showed that there was no direct link between 5-azaC-induced genomic DNA demethylation and DNA strand breaks. Therefore, these two toxicological endpoints can be considered to be independent of one another.

(2) Our data showed significantly different genotoxic and epigenotoxic effects of the tested molecules, with PK9323 being the most active in inducing molecular modifications. In fact, all the biological and epigenetic endpoints, at both the molecular and nuclear/cellular level, clearly showed that this specific carbazole derivative possesses a high (epi)genotoxic potency, as demonstrated by the highly significant differences in the γH2AX phosphorylation, cell viability, Comet and MeSAP-PCR assays relative to the control and the other tested carbazoles. This is the first description of the genotoxicity of this newly synthesized molecule and, more generally, the first description of combined effects, both genomic and epigenomic, of a carbazole derivative.

(3) Compound PK9320 might be labeled as genotoxically inactive, given that it was not found to affect the level of histone phosphorylation; however, it is noteworthy that it caused a drastic decrease in tubulin levels, indicative of cell death or inhibition of cell growth. This result prompted us to examine the toxicity of this specific molecule from different angles by probing its endocellular/endonuclear and epigenetic action, because carbazole derivatives have been reported as non-toxic mainly when tested at the molecular level, but highly genotoxic when submitted to other tests, including the Comet assay [12]. Consistent with these observations, we found that PK9320, at 10 µM concentration, was able to reduce cell numbers in a highly significant way. Moreover, it was unable to cause significant damage to nuclear DNA, as revealed by the Comet assay, at both 2.2 and 10 µM concentrations, but induced a statistically significant reduction of genomic DNA methylation in both experimental conditions. PK9320, in summary, at the concentration used in this study, appears to be a carbazole derivative endowed with a certain capability to exert DNA damage, but mainly coming from its epigenomic modifying action at least with regard to DNA methylation: an “epidrug” as defined by Ghasemi [31], which could be better renamed as “pure epidrug”, given its specificity of action.

(4) Finally, PK083, a molecule structurally similar to PK9320 and PK9323, has shown surprisingly attenuated, and in some cases even opposite, effects in almost all the analyses performed in this study. In fact, this compound exerted a non-genotoxic effect when assayed by phospho-γH2AX test and a protective effect on basal DNA damage, which was highly significant at both administered concentrations with respect to the control. Moreover, MeSAP-PCR data revealed that PK083 displayed a non-significant demethylating potency on the CpG sites of genomic DNA, lower than that of 5-azaC. It may be concluded that PK083 is a non-(epi)genotoxic molecule with a similar behavior to that reported for ellipticine [32], a molecule with a tumor cell growth inhibitory ability, judged as non-genotoxic by γH2AX assay. In our experimental system, the sole activity performed by PK083 that can be considered toxic was the reduction of MCF-7 cell number when administered at 10 µM concentration.

Although there are still several unanswered questions that our study did not address, such as the effect of carbazoles on histone acetylation/deacetylation balances, miRNA variations and DNA methylation at the gene level, overall our data show that small modifications in the substitution patterns of carbazoles can have profound effects on their intrinsic genotoxic and epigenetic properties, which may be exploited for their use as anticancer agents and probe molecules.

## 4. Materials and Methods

### 4.1. Cell Culture and Treatments

The MCF-7 breast tumor cell line was cultured in high glucose–DMEM medium plus 10% foetal calf serum (FCS; ThermoFisher, Waltham, MA, USA), 100 U/mL penicillin, 100 μg/mL streptomycin, and 2.5 mg/L amphotericin B (Invitrogen, Carlsbad, CA, USA), at 37 °C in a 5% CO_2_ atmosphere. The cells were detached from flasks with 0.05% trypsin-EDTA (Invitrogen), counted, and plated at the necessary density for treatment after achieving 60−80% confluency. PK083, PK9320 and PK9323 were synthesized as reported by Bauer et al. [15], dissolved in dimethyl sulfoxide (DMSO) firstly at 330 and 1500 mM stock concentrations and then in 0.33 and 0.15 mM work concentrations, aliquoted in several tubes to avoid freezing–thawing cycles and stored at −20 °C. The biological assays were carried out for 20 h in the presence of the respective compound at the concentrations indicated in the specific paragraphs; the working concentrations were obtained by dilution of either stock solution with a constant volume of DMSO. Negative controls were prepared by exposing cells to the same volume of DMSO vehicle only for 20 h. No significant difference between untreated cells and DMSO-treated ones was found in relation to each biological endpoint investigated. As a positive control for the epigenomic studies, cells were treated with a 10 µM concentration of the DNA methyltransferase inhibitor 5-azaC for 48 h [25].

### 4.2. Morphological Observations and Cell Number Evaluation

MCF-7 cells were plated at a density of 1.0 × 10^6^ cells/mL in 60-mm Petri dishes, cultured in control conditions or in the presence of either PK083, PK9320 or PK9323 and observed under an inverted OPTIKA phase-contrast microscope (Ponteranica, Italy) at the end of the treatment. At least 50 microscopic fields were examined in three independent experiments. Cell numbers were measured by direct counting in a Bürker chamber after trypsinization [33].

### 4.3. DNA Damage Assays

#### 4.3.1. Western Blot for Phospho-γH2AX Histone

MCF-7 breast cancer cells were seeded at a density of 1.0 × 10^5^ cells/well in a 12-well plate and allowed to settle for 48 h before exposure to PK083, PK9320, or DMSO. At the end of the treatments, the cells were washed with phosphate-buffered saline and lysed using 1x SDS-loading buffer containing β-mercaptoethanol (5x buffer: 0.25% *w/v* bromophenol blue, 0.5 M DTT (dithiothreitol), 50% *v/v* glycerol, 10% *v/v* SDS (sodium dodecyl sulphate) and 0.25M Tris-HCl, pH 6.8). The lysates were sonicated and clarified by centrifugation at 13,000 r.p.m. before being loaded into 12% SDS-polyacrylamide gels. Proteins were separated by SDS-PAGE at 200 V for 35 min. Western blot analysis of phospho-γH2AX histone and the loading control α-tubulin was accomplished through transfer of protein onto PVDF membranes using the Trans-Blot Turbo System (Bio-Rad, Hercules, CA, USA). Mouse monoclonal anti-α-tubulin (DM1A) was purchased from Abcam (Cambridge, UK) and mouse anti-phospho-histone H2AX (Ser139) (JBW301) was purchased from Millipore (Darmstadt, Germany). Secondary antibodies were HRP-conjugated, polyclonal goat-derived antibodies against mouse and rabbit (Dako, Agilent Technologies, Santa Clara, CA, USA). After blotting and immunoreactions, protein bands were detected with a chemiluminescent Western blotting substrate (SuperSignal West Femto, ThermoFisher) and the ImageQuant LAS 4000 system (GE Healthcare, Chicago, IL, USA). Protein bands were quantified by densitometry using ImageJ 1.50C and phospho-histone H2AX bands were normalized to the α-tubulin control band. DNA damage was reported as the percent of normalized phospho-histone H2AX. DNA damage assays were repeated in triplicate and the half maximal effective concentration (EC_50_) value for PK9323 compound compared with that of cisplatin as a reference DNA lesion-inducing drug using the non-linear regression module of Prism 6.0 (GraphPad, San Diego, CA, USA). In light of the results obtained from the dose–response assays with PK9323, the following experiments were carried out for 20 h in the presence of either 2.2 (PK9323 EC_50_) or 10 μM (maximum concentration tested) of either of the three compounds; both working concentrations were obtained by dilution of either stock solution with a constant volume of DMSO. Negative controls were prepared by exposing cells to the same volume of DMSO vehicle only for 20 h.

#### 4.3.2. Alkaline Comet Assay

The extent of the DNA damage in MCF-7 cells by PK083, PK9320 and PK9323 was examined via single cell gel electrophoresis with the OxiSelect Comet assay kit (Cell Biolabs, San Diego, CA, USA) as previously described [34,35]. Essentially, control and treated cells were mechanically detached, resuspended at a density of 1.0 × 10^5^/mL in Ca^++^/Mg^++^-free phosphate-buffered saline and mixed with Comet agarose. The samples were then loaded onto the OxiSelect Comet slides, allowed to gel, and treated with the lysis buffer followed by an alkaline solution allowing DNA unwinding and denaturation. The damaged DNA exhibiting both single- and double-strand breaks was separated from the intact fraction through alkaline gel electrophoresis and, at the end of the run, the slides were fixed, air-dried, and stained with Vista Green DNA or Gel red (Biotium, Fremont, CA, USA) Dye. At least 50 nuclei were selected randomly per experiment, observed and photographed under a Nikon Microphot SA fluorescence microscope and the olive tail moment (OTM) was evaluated with the CASP Version 1.2.3b1 software (Sourceforge, Diceholdings Inc., New York, NY, USA).

### 4.4. DNA Extraction and Methylation-Sensitive Arbitrarily Primed Polymerase Chain Reaction (MeSAP PCR)

Extraction of the genomic DNA of control and treated MCF-7 cells was performed using the PureLink Genomic DNA Kit (Invitrogen) according to the manufacturer’s instructions, and the extracted DNA was quantitated using a NanoDrop^®^ ND-1000 spectrophotometer (ThermoFisher), as previously described [36].

In order to evaluate the modulatory effects of PK083, PK9320 and PK9323 on the genome-wide DNA methylation level, MeSAP-PCR experiments were carried out as previously reported [37,38]. Essentially, single-digested DNA (SDD) samples were obtained by treating the genomic DNA with *Afa*I restriction endonuclease (ThermoFisher) whose cleavage site is (GT*AC). Subsequently, in order to obtain double-digested DNA (DDD), samples aliquots of SDD were further treated with the methylation-sensitive *Hpa*II restriction endonuclease (ThermoFisher), ineffective in cleaving the DNA when its recognition site (CG*GG) contains a methylated cytosine. An arbitrarily primed two-step PCR experiment was performed to amplify both SDD and DDD samples. The first PCR step (cycle profile: 94 °C for 5 min, four cycles at 94 °C for 30 s, 40 °C for 60 s and 72 °C for 90 s) was carried out in the presence of a 21-mer arbitrary primer (5′-AACTGAAGCAGTGGCCTCGCG-3′) in low stringency conditions, i.e., a permissive annealing temperature and a high salt and primer concentration. This allows for the best matches between the arbitrary primer and the genomic CpG sites, due to the complementary 3′ end of the former one. The second highly stringent PCR step (cycle profile: 94 °C for 1 min, four cycles at 60 °C for 1 min and 72 °C for 2 min) was carried out in continuity, and, at the end, the amplified DNA was separated through a non-denaturing 6% polyacrylamide gel electrophoresis, stained with Gel Red nucleic acid gel stain (Biotium, Hayward, CA, USA), and analyzed with SigmaGel v.1.0 image analysis software (SPSS, Chicago, IL, USA).

### 4.5. Pharmacokinetic (PK) Studies

PK studies were carried out as outlined previously [39,40]. A detailed description of the protocols can be found in the supporting information file.

### 4.6. Statistics

Data are presented as mean ± SEM (SD in Figure 3) from three independent experiments. A Student’s *t* test was used and *p* < 0.05 was taken as the minimum level of statistical significance between treated and control samples. The IQR values were calculated and represented as a black line in the histograms.

## 5. Conclusions

In conclusion, we have studied the genotoxic and epigenotoxic properties of three carbazole-derived molecules and demonstrated that, despite their similar structure, they have very different biological activities, in particular towards the DNA molecule as a target.

**Scheme 1:**
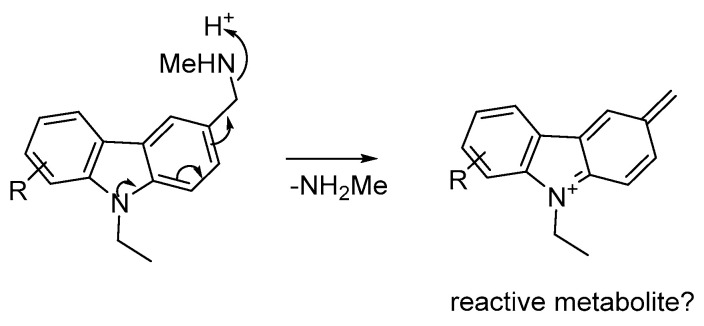
Potential amine loss in carbazoles observed in high-resolution electrospray ionization mass spectrometry (ESI-HRMS).

The physicochemical properties of this carbazole series warrant some comments. In the mass spectra of these analogues, we sometimes observed species with a reduced mass that would be consistent with the loss of the amine side group and the formation of a reactive iminium-methide species (Scheme 1; [15,41]). Given that the NHMe group is an important interacting moiety for carbazoles in the p53-Y220C cavity (it forms an H-bond with a D228 backbone carbonyl group as reported by [41]), such a reduction of binding affinity would reduce their mutant p53 reactivation potential and possibly lead to off-target effects. This aspect may be considered when designing the next generation of carbazole-based Y220C mutant stabilizers to incorporate an interacting moiety that cannot result in an elimination reaction, for example, by introducing an additional methylene group between the carbazole ring and the amine group. However, so far there is no evidence that this elimination occurs in cells.

Apart from expanding the biological knowledge of the effects of specific carbazoles on cellular genome status, the molecular aspects put in evidence in our study may provide promising targets for anti-cancer therapeutic interventions. In perspective, we can define these molecules, and **PK9320** and **PK9323** to a greater extent, as pharmacologically active substances and, although further investigation will be necessary, as eligible candidates for “anticancer drugs” and “anticancer epi-drugs” on human breast adenocarcinoma cells. In particular, the lower dose of both molecules used in our experimental conditions appears to be the most promising, because the higher dose could be so genotoxic to operate a dangerous clonal selection toward the development of clones more able to repair DNA breakages and thus more aggressive. In addition, the property of the **PK083** molecule, which according to our data is even corrective of the basal DNA damage, will be worth investigating, thereby opening new potential scenarios for its future biomedical applications.

## Data Availability

The data that support the findings of this study are available from the corresponding author upon reasonable request.

## Supplementary Materials

The following is available online at https://www.mdpi.com/article/10.3390/ijms22073410/s1, Figure S1: Optical densities of the Western blot bands; Figure S2: Phase-contrast micrographs of control and treated cells; Table S1: Variation of DNA band pattern as index of genomic demethylation; Protocols for PK studies; Table S2: Results from PK studies.
