# Supplementary material for: Genotoxicity and Epigenotoxicity of Carbazole-Derived Molecules on MCF-7 Breast Cancer Cells
Source: Int J Mol Sci. 2021 Mar 26;22(7):3410. doi: 10.3390/ijms22073410 (PMC8038095; doi:10.3390/ijms22073410)
Supplement: Supplementary file 1 [file ijms-22-03410-s001.zip › ijms-1148277-supplementary.docx]

***Supplementary***

**Genotoxicity and epigenotoxicity of carbazole-derived molecules on MCF-7 breast cancer cells**

**Fig. S1**. Optical densities of the Western blot bands shown in figs. 2 and 3

| 1 μM | | | | |
| --- | --- | --- | --- | --- |
|  | pH2AX |  | tub |  |
|  | 1 | 2 | 1 | 2 |
| DMSO | 1143.197 | 825 | 991.676 | 1294 |
| PK083 | 801.538 | 699.679 | 1278.726 | 1177.74 |
| PK9320 | 684.404 | 588.642 | 1390.687 | 1301.788 |
| PK9323 | 786.345 | 687.233 | 1023.452 | 938.806 |
| blank | 342.402 | 338.982 | 360.639 | 271.472 |
| 10 μM | | | | |
|  | pH2AX |  | tub |  |
|  | 1 | 2 | 1 | 2 |
| DMSO | 1045.36 | 715.06 | 916.521 | 1220.359 |
| PK083 | 842.048 | 738.957 | 1116.425 | 1043.135 |
| PK9320 | 757.778 | 665.781 | 522.484 | 448.662 |
| PK9323 | 1199.825 | 1105.789 | 396.18 | 321.129 |
| 1 μM | | | | |
| Corrected | pH2AX |  | tub |  |
|  | 1 | 2 | 1 | 2 |
| DMSO | 800.795 | 486.018 | 631.037 | 1022.528 |
| PK083 | 459.136 | 360.697 | 918.087 | 906.268 |
| PK9320 | 342.002 | 249.66 | 1030.048 | 1030.316 |
| PK9323 | 443.943 | 348.251 | 662.813 | 667.334 |
|  |  |  |  |  |
| 10 μM | | | | |
|  | pH2AX |  | tub |  |
|  | 1 | 2 | 1 | 2 |
| DMSO | 702.958 | 376.078 | 555.882 | 948.887 |
| PK083 | 499.646 | 399.975 | 755.786 | 771.663 |
| PK9320 | 415.376 | 326.799 | 161.845 | 177.19 |
| PK9323 | 857.423 | 766.807 | 35.541 | 49.657 |

normalised to tub

| 1 μM | | | |
| --- | --- | --- | --- |
| H2AX/tub | |  |  |
| 1 | 2 | avg | stdv |
| 1.26901434 | 0.47531021 | 0.87216227 | 0.39685206 |
| 0.50010075 | 0.39800258 | 0.44905167 | 0.05104909 |
| 0.3320253 | 0.24231401 | 0.28716966 | 0.04485565 |
| 0.6697862 | 0.52185412 | 0.59582016 | 0.07396604 |

| 10 μM | | | |
| --- | --- | --- | --- |
| H2AX/tub | | tub | |
| 1 | 2 | avg | stdv |
| 1.26458133 | 0.39633592 | 0.83045862 | 0.43412271 |
| 0.66109454 | 0.5183286 | 0.58971157 | 0.07138297 |
| 2.56650499 | 1.84434223 | 2.20542361 | 0.36108138 |
| 24.124898 | 15.4420726 | 19.7834853 | 4.34141269 |

Fig. 3

| PK9323 |  |  |  |  |  |  |
| --- | --- | --- | --- | --- | --- | --- |
| pH2AX | |  | pH2AX bck correct | | |  |
| Conc. μM | 1 | 2 | 3 | 1 | 2 | 3 |
| Cisplatin (4 μM) | 1073.838 | 515.47 | 928.58 | 838.838 | 280.47 | 693.58 |
| 0 | 415.397 | 356.246 | 425.476 | 180.397 | 121.246 | 190.476 |
| 1.25 | 571.799 | 425.978 | 518.824 | 336.799 | 190.978 | 283.824 |
| 2.5 | 909.395 | 724.496 | 897.506 | 674.395 | 489.496 | 662.506 |
| 5 | 1085.255 | 1095.347 | 1134.332 | 850.255 | 860.347 | 899.332 |
| 10 | 857.842 | 868.487 | 931.323 | 622.842 | 633.487 | 696.323 |
| Tub |  |  | Tub bck correct | |  |  |
| Conc. μM | 1 | 2 | 3 | 1 | 2 | 3 |
| Cisplatin (4 μM) | 1454.003 | 1105.768 | 1643.718 | 1242.003 | 893.768 | 1431.718 |
| 0 | 1299.762 | 1319.784 | 1714.85 | 1087.762 | 1107.784 | 1502.85 |
| 1.25 | 1112.4 | 1197.997 | 1323.394 | 900.4 | 985.997 | 1111.394 |
| 2.5 | 1110.009 | 1087.853 | 1268.073 | 898.009 | 875.853 | 1056.073 |
| 5 | 1029.73 | 1019.336 | 1150.782 | 817.73 | 807.336 | 938.782 |
| 10 | 902.02 | 849.239 | 1078.414 | 690.02 | 637.239 | 866.414 |

**Figure 2.** Low (A-G) and high magnification (A+-G+) phase contrast micrographs of DMSO-treated control MCF-7 cells (A,A+) and parallel cultures exposed to 2.2 and 10 μM **PK083** (B,B+ and E,E+), **PK9320** (C,C+ and F,F+) or **PK9323** (D,D+ and G,G+) for 20 h. Black bar = 5 µm; Red bar = 25 µm. Microscope magnification = 20X (A-G) and 100X (A+-G+). The high magnification photos allow to better assess cell morphology.

***Table 1.*** Variation of DNA band pattern as index of genomic demethylation*.*

| **Treatment**  **(concentration)** | **DNA band pattern variation**  **appearing/ attenuation/ disappearing intensification** | **Total**  **variations** |
| --- | --- | --- |
| DMSO | 8 6 | 14 |
| 5-azaC | 17 4 | 21 |
| **PK083** (2.2 μM) | 14 4 | 18 |
| **PK083** (10 μM) | 14 3 | 17 |
| **PK9320** (2.2 μM) | 18 3 | 21 |
| **PK9320** (10 μM) | 12 10 | 22 |
| **PK9323** (2.2 μM) | 27 4 | 31 |
| **PK9323** (10 μM) | 20 3 | 23 |

Protocols for PK studies

Solubility

This procedure was designed to determine the solubility of a test compound in buffer at a set pH in a 96-well plate format. The test compound was quantified at up to 6 time points in triplicate by UV-VIS spectroscopy at 3, 10, 25, 50, 75 and 100 μM (6 concentrations) with a final DMSO concentration of 1%. For the 96 well-solubility assay a serial dilution of compound was made up in DMSO and added to the test plate. Buffer was added and the plate agitated at room temperature for up to 16 h. Blanks of buffer and DMSO were run in parallel (16 per plate). The sample is read in a spectrophotometer at 650 nm and the absorbance measured. Two standards were used. Pyrene was used as a positive control and aspirin as a negative control.

Microsomal stability test

The stability of a test compound in mouse microsomes was routinely performed in a 96-well plate format. The test compound was quantified at up to 6 time points in duplicate by HPLC-MS/MS analysis at a test concentration of 5 μM with a final DMSO concentration of 0.2%. The assay buffer was pre-warmed with compound and cofactor solution (NADP, G6P and G6PDH) to 37°C in a thermomixer. Microsomes were added to the reaction mixture and aliquots were removed at specified time points and transferred to acetonitrile in a protein precipitation plate at typically 0, 0.08, 0.25, 0.5, 0.75 and 1 h respectively, pulled through under vacuum and the aliquots dried under nitrogen and re-suspended in mobile phase for HPLCMS/MS analysis. Verapamil was used as a positive control and controls containing compound and microsomes only (no cofactors) were used to look for any non-CYP450 degradation. Samples were analysed by HPLC-MS/MS using selected reaction monitoring. The HPLC system consisted of a binary LC pump with autosampler, a C-18 column, and a gradient. **Data analysis**: peak areas corresponding to the test compound were recorded. The compound remaining (%) was calculated by comparing the peak area at each time point to time zero. The half-life was calculated from the slope of the initial linear range of the logarithmic curve of compound remaining (%) vs. time, assuming first order kinetics and the apparent CLint calculated.

Plasma protein binding (PPB) studies

This procedure was designed to determine the binding of a test compound to plasma protein from mouse in a 96-well plate format. The test compound was quantified in triplicate by HPLC-MS/MS analysis. The test concentration used was 10 μM with a final DMSO concentration of 0.1%. For the 96-well plasma protein binding assay, plasma was pre-warmed to 37°C and placed into a Rapid Equilibration Device in a thermomixer along with compound. Buffer (PBS) was placed in the other incubation chamber and the thermomixer agitated for up to 4h at 37ºC. An aliquot from both the plasma and buffer chamber were transferred to acetonitrile in a protein precipitation plate at the end of the incubation period, pulled through under vacuum and the aliquots dried under nitrogen and resuspended in mobile phase for HPLC-MS/MS analysis. A concentration curve of the compound was extracted in the same matrix. Verapamil was used as a positive control. Samples were analysed by HPLC-MS/MS using selected reaction monitoring. The HPLC system consisted of a binary LC pump with autosampler, a C-18 column, and a gradient. **Data analysis**: Peak areas corresponding to the test compound were recorded and quantified against the standard curve of the same compound. The % compound bound was calculated from the compound concentrations in the plasma and buffer chambers.

Cytotoxicity assay (according to [40])

HepG2 liver cancer cells were plated at 5,000 cells per well of a 96 well-plate in growth media containing 10% Foetal Bovine Serum (FBS). After 24 h compounds were added to the cells at increasing concentrations in triplicate tests. Cell plates were incubated at 37°C, 5% CO_2_ over a 72 hour period and assayed for ATP levels as indicators of cell health using Promega CellTitre-Glo^®^ assay kit (Madison, WI, USA) and luminescence read on SpectraMax iD5 (Molecular Devices, San Jose, CA, USA). **Data analysis:** Data were normalised to % of control (no compound) and plotted against the compound concentration to determine an EC_50_ value.

**Table 2.** Results from PK studies.

| **Compound** | **HepG2 Cytotox EC_50_ (µM) MEAN** | **Limit of Solubility pH 7 (µM) MEAN** |  |  | **Microsomes (mouse) Clint (µL/min/mg) MEAN** |  | **Microsomes (mouse) T½ (min) MEAN** | **PPB (mouse) % Bound MEAN** | **PPB (mouse) % Unbound MEAN** |  |  |
| --- | --- | --- | --- | --- | --- | --- | --- | --- | --- | --- | --- |
| **PK083** | 23 | >100 |  |  | 30 |  | 45.6 | 91 | 9 |  |  |
| **PK9320**  **PK9323** | 4.8  1.1 | >100  >100 |  |  | 56  18.5 |  | 24.8  74.9 | 99.8  97 | 0.2  3 |  |  |
